# Thermal Transmission through Existing Building Enclosures: Destructive Monitoring in Intermediate Layers versus Non-Destructive Monitoring with Sensors on Surfaces

**DOI:** 10.3390/s17122848

**Published:** 2017-12-08

**Authors:** Víctor Echarri, Almudena Espinosa, Carlos Rizo

**Affiliations:** 1 Department of Architectural Constructions, University of Alicante, Carretera San Vicente del Raspeig, s/n, 03690 San Vicente del Raspeig, Spain; carlosrm@ua.es; 2 Department of Achitecture, University of Zaragoza, Calle María de Luna, 3, 50018 Zaragoza, Spain; almudenaef@unizar.es

**Keywords:** thermal transmittance, building monitoring, data correction algorithm, Raspberry Pi, interior comfort, thermal inertia, annual energy demand

## Abstract

Opaque enclosures of buildings play an essential role in the level of comfort experienced indoors and annual energy demand. The impact of solar radiation and thermal inertia of the materials that make up the multi-layer enclosures substantially modify thermal transmittance behaviour of the enclosures. This dynamic form of heat transfer, additionally affected by indoor HVAC systems, has a substantial effect on the parameters that define comfort. It also has an impact on energy demand within a daily cycle as well as throughout a one-year use cycle. This study describes the destructive monitoring of an existing block of flats located in Alicante. Once the enclosure was opened, sensors of temperature (PT100), air velocity, and relative humidity were located in the different layers of the enclosure, as well as in the interior and exterior surfaces. A pyranometer was also installed to measure solar radiation levels. A temperature data correction algorithm was drawn up to address irregularities produced in the enclosure. The algorithm was applied using a Raspberry Pi processor in the data collection system. The comparative results of temperature gradients versus non-destructive monitoring systems are presented, providing measures of the transmittance value, surface temperatures and indoor and outdoor air temperatures. This remote sensing system can be used in future studies to quantify and compare the energy savings of different enclosure construction solutions.

## 1. Introduction

It is well known that energy consumption of buildings accounts for approximately 40% of total energy consumption in the European Union (EU) [1,2,3]. Furthermore, environmental impacts deriving from their construction, use, maintenance, as well as Construction and Demolition Waste (CDW), are considerable. A major line of research in the field of energy consumption focuses on the enclosures of building façades [4,5,6]. Internal–external heat transfer through these enclosure components depends on a series of factors: the physical parameters of the materials they are made with; their air permeability [7]; the quality of the construction work; their vapour permeability; and thermal bridge effects produced by thermal insulation discontinuities [8,9]. Furthermore, heat transfer has adverse effects on users’ thermal comfort, leading to further energy consumption specifically aimed at reaching the comfort levels required in these buildings [10,11].

Fourier’s law is applicable to parallel layers of unlimited surface materials in a static regime of indoor *T_i_* and outdoor *T_o_* air temperatures. The law establishes a multi-layer enclosure’s temperature gradient when a thermal gap exists between outdoor and indoor air temperatures. Heat transfer by conduction or by convection comes into play according to values of resistance to the passage of heat, or to surface thermal resistance resulting from generated convection currents.

$$U-value=\frac{1}{R_{T}}=\frac{1}{\frac{1}{hi}+\sum_{0}^{n}\frac{e_{i}}{\lambda_{i}}+\frac{1}{he}},$$

where:

- $R_{T}$ Total heat thermal resistance (m2 °CW)
- $\frac{1}{hi}$ Outdoor surface thermal resistance (m2 °CW)
- $\frac{1}{he}$ Indoor surface thermal resistance (m2 °CW)
- $e_{i}$ Own layer thickness (m)
- $\lambda_{i}$ Thermal conductivity of the material layer (Wm2 °C)

The *U-value* is the inverse of the total resistance value of the enclosure heat transfer *R_T_*, and represents the value, in watts, of the heat flow produced between outdoor and indoor environments, per unit area (m^2^) and for each degree of temperature difference *T_o_ − T_i_*. This parameter, in which the insulation layer plays an essential role, is used when designing buildings to quantify annual energy demand, thermal comfort of the indoor environment, thermal loads for the design and sizing of the air conditioning systems as well as for assessing the risk of interstitial condensation that can potentially cause damage.

**Figure 1:**
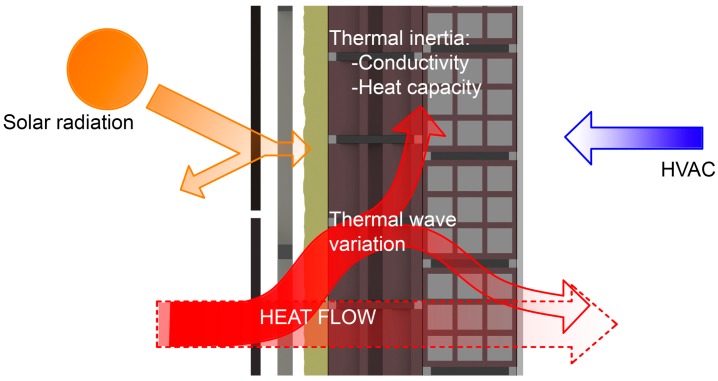
The dynamic process of heat transmission through enclosures.

However, this law is valid only for ideal situations. Walls have limited dimensions and present discontinuities due to metalwork, woodwork, and glazing. Thermal bridges also exist due to construction requirements, etc. [12,13]. Furthermore, different layers are often made up of various types of materials with different physical parameters regarding thermal resistance values [14]. In addition, outdoor air conditions differ according to solar radiation, wind speed, water vapour pressure, etc. Solar radiation frequently affects outer surfaces, provoking substantial increases in temperature [15]. Additionally, if we look at the indoor environment, different thermal loads and conditioning systems alter the physical parameters of relative humidity, indoor air temperature, and wall surface temperatures. To finish, Fourier’s linear process is altered by variations in the thermal inertia of enclosure materials over time. These variations are due to the thermal gap between indoor and outdoor air temperature, solar radiation affecting the exterior surface, or indoor air conditioning systems, that all change according to different moments in time. Heat flow can thus be defined as a dynamic and complex process [16] (Figure 1).

This study aims at examining the thermal behaviour of a residential building’s enclosure located in Alicante. The enclosure was monitored over the complete cycle of year 2013, in order to be able to adequately evaluate its thermal behaviour in the different stations, and to analyse in a comparative way the effect of the thermal inertia of its materials and the incident solar radiation. Surface temperature sensors PT100 were fixed on all the interior layers of the enclosure. The system used to pierce the layers produced a perforation of approximately 0.40 m in diameter in the lower right section. The sensors were thus introduced into all five layers that made up the wall. Subsequently, these layers were sealed with materials that were similar to the original ones. Because of the difficulty in executing this construction work, certain discontinuities in the construction materials occurred. The sensors enabled drawing a graph representing thermal gradient curves throughout the year, as well as variations in the dynamic regime. Given there were differences between the sensors’ temperature measurements and those that would have been recorded had the wall not been perforated, a mathematical model was made to correct Information Technologies (IT). These types of corrections have been previously used by experts in various studies [17,18,19]. Correction factors were calculated based on the position of the open hole in the wall, its simplified diameter, and the thermal *λ* and *λ*_1_ conductivities of original and repair materials. The temperatures originally obtained were thus automatically corrected. Current advances in the integration of components and multi-agent technologies applied to network sensors have led to a new model of multisensory processing designed for monitoring climate behaviour, as described in this article.

**Figure 2:**
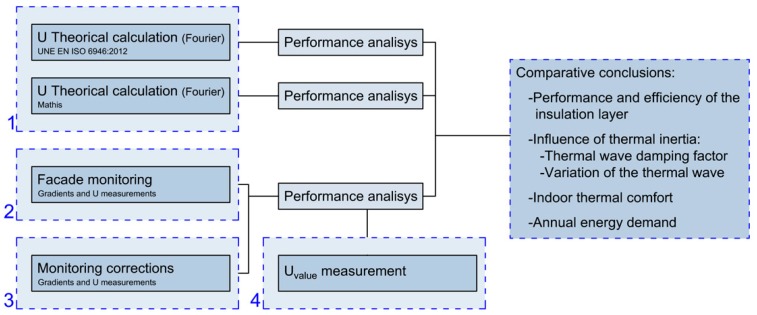
Design of the research methodology process.

The research methodology is shown in Figure 2. The first step consisted in examining the thermal behaviour of the enclosure in a steady state according to Fourier’s law, applying theoretical values of *λ* thermal conductivities according to the UNE EN ISO 6946:2012 standard. Secondly, the same analysis was performed based on thermal conductivity measurements values using Mathis TCi equipment. Thirdly, the monitoring data were analysed, as well as the monitoring data corrected by the mathematical model, once the enclosure had been pierced and repaired. These results were analysed comparatively, mainly through graphs, detecting temperature variations in the daily cycle due to the materials’ thermal inertia and solar radiation. Finally, measurements of *U-values* were made both for the original enclosure and for the repaired enclosure.

By comparing these graphs obtained by monitoring with graphs based on Fourier’s theoretical behaviour in static conditions together with the measurements results of *U-values*, it was possible to quantify the efficiency of the insulation layer of polyurethane foam type II. This also enabled identifying the effect of some parameters affecting thermal transmission, such as effusivity or thermal conductivity of materials. In future work, the thermal wave’s damping factor and lag could be quantified in different seasons of the year. A thermal wave was produced by the thermal gap between indoor and outdoor air temperatures. The wave increased in summer due to solar radiation effects on the ventilated façade but was reduced, for example, thanks to the materials’ thermal inertia. The behaviour of the ventilated façade in its capacity to dissipate the heat absorbed by the porcelain stoneware was also analysed. Much of this heat was dissipated through the cavity’s convection air currents, though this caused the outer face of the thermal insulator to heat significantly due to radiation [20,21]. The results of this study can be used in future building construction works as they allow for drawing up corrections when building enclosures. Furthermore, our results enable calculating the actual annual energy demand of existing buildings.

## 2. Description of the Building and Its Northwest Enclosure

**Figure 3:**
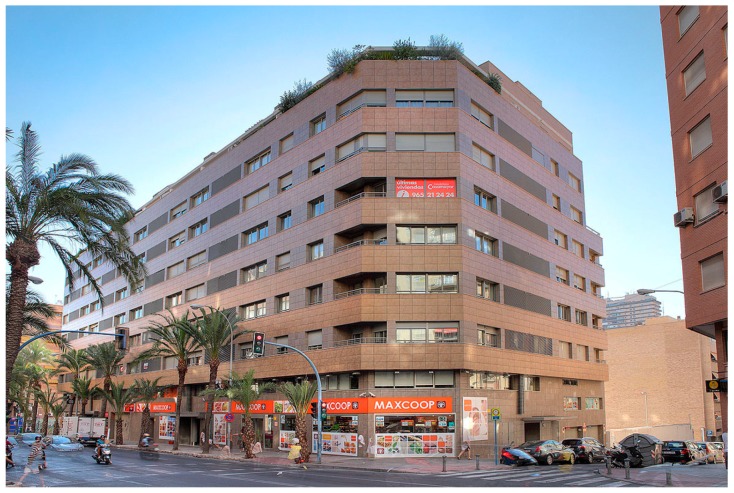
Building façade. North façade and west façade.

The building under study was located in the urban area of Alicante city (Figure 3). This corner building has a façade giving onto Benito Pérez Galdós Avenue and another giving onto Catedrático Ferre Vidiella Street. The building houses 69 flats with two, three or four bedrooms, garages and storage rooms. It has eight different types of homes (flats A–F, penthouse types N–P). It is a corner building between party walls, with a ground floor, six more floors and a loft. There are five vertical communication nuclei: four of them with direct access to Benito Pérez Galdós Avenue and the fifth with direct access to Catedrático Ferre Vidiella Street.

**Figure 4:**
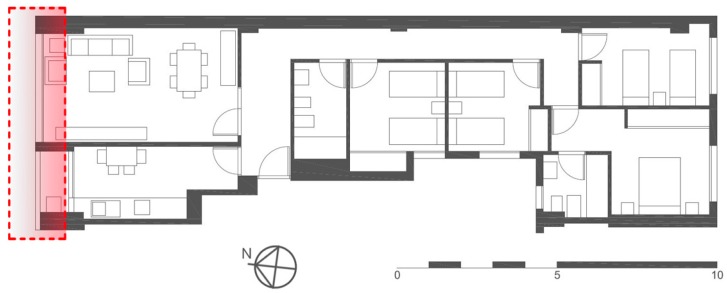
General floor view of type C flat.

The housing under study corresponded to the building’s type C flat. It is located on the third floor, with north and south facades. It consists of two bathrooms, four bedrooms (two bedrooms giving onto the interior patio of the building measuring 4 m^2^ × 4 m^2^ and two bedrooms giving onto the block’s interior courtyard), a living room and kitchen oriented towards the north façade giving onto Benito Pérez Galdós Avenue (Figure 4). None of the flat boundaries give onto the party walls so all of the flat’s partitions are bordered by other flats in the same building.

The layers and technical characteristics of the opaque enclosure of the northwest façade are described in Table 1. Thermal resistance and *U-value* are specified in accordance with the current Spanish Technical Building Regulations (herein referred to using its Spanish acronym, CTE):

The ventilated façade has a layer of perforated bricks, with a substructure of aluminium quadrangular pipes and beams, a continuous insulation layer of projected polyurethane foam 3 cm thick, according to the architecture project, lined with 10 mm thick pieces of porcelain stoneware.

The air conditioning system consists of a VRV split inverter system. The condensing machine is located on the roof of the building, and an evaporator of 3.200 W is fixed in the suspended ceiling of the general bathroom. Conditioned air is distributed via rectangular fibreglass ducts covered in aluminium foil, which run down the corridor and propel the flow of air through double deflection ventilation grilles, based on a flow-regulating device. Flow rate propulsion is regulated specifically for each room, and the valve has a start–stop mechanism based on the room’s thermostat.

## 3. Monitoring of the Northeast Façade

The monitoring equipment was installed a few years after the construction of the building. To be able to fix some of the surface temperature sensors, a quasi-circular surface of around 40 cm in diameter had to be destroyed, since four layers of the enclosure had to be broken. The hole made in the insulation layer had a diameter of about 25 cm. The apparatus installed included sensors for measuring temperature as well as relative humidity of outdoor and indoor air, surface temperature in the different layers of the enclosure, air speed indoors and in the external cavity, and solar radiation using a pyranometer located 1 m from the façade (Table 2). Temperature sensors were placed in each of the layers of the façade’s enclosure. A humidity sensor was placed in the layers of the enclosure where interstitial condensation could occur.

### 3.1. Description of the System: Connection Flow Chart

**Figure 5:**
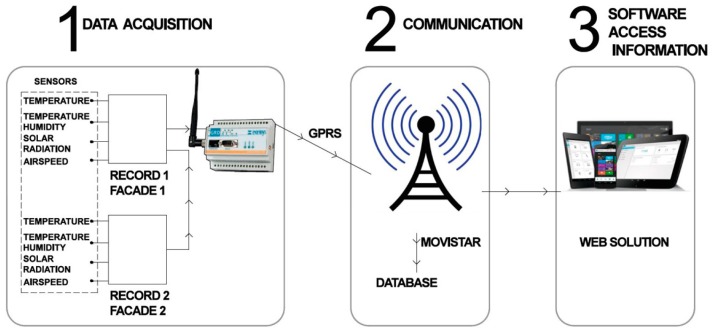
Connection flow chart.

The monitoring system consists of the probes described above, placed at the strategic points of the building’s two enclosures, determined according to the physical composition of the glazing and front slabs. Data collected by the sensors are sent to a data logger (analyser), which transmits a signal via GPRS to a database. This information is accessible from a web platform (Figure 5).

#### 3.1.1. Description of the Analyser

**Figure 6:**
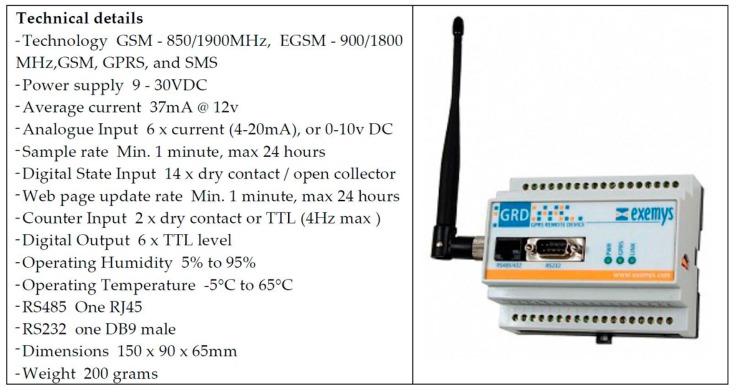
Technical file of the Data Logger.

The GSM/GPRS data logger GRD allows data to be recorded via analogue or digital channels. Through its GSM/GPRS modem, the data are sent to a server and can be consulted on the web in graph or table format (Figure 6). They can also be downloaded as CSV files.

#### 3.1.2. Description of the Sensors

**Figure 7:**
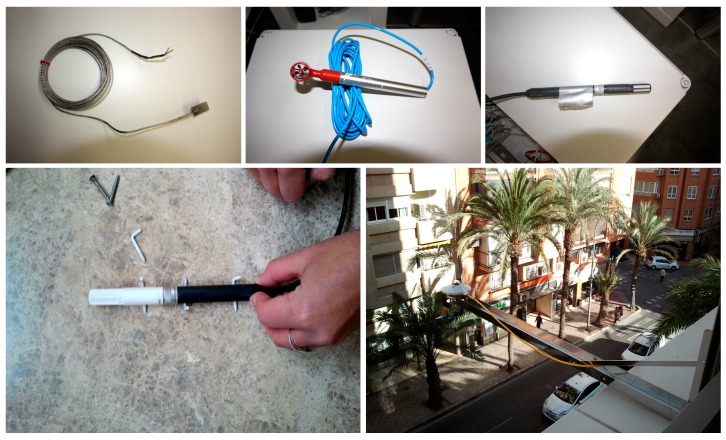
Sensors for surface temperature, anemometer and environment temperature; and pyranometer.

Environmental variables are individually monitored for each enclosure construction solution (Figure 7). Records were made of:

1. Air temperature and relative humidity, both inside and outside the buildings: via temperature and humidity probes (thermo-hygrometers).
2. Surface temperature of building materials: using temperature probes placed in the interior and exterior layers of the enclosures.
3. Relative humidity inside the enclosures: by means of humidity probes (hygrometers).
4. Solar radiation outside the enclosures: using a pyranometer sensor.
5. Air velocity inside the enclosures that contain ventilated air cavities: by anemometer probe.

The technical characteristics of the data logger and sensors are as follows:

- GPRS RTU, one RS232/385 port, two pulse inputs, 14 digital inputs, six Open Collector Digital Outputs, six 0–10 V Analog Inputs`. Modbus master.
- Standard Hygroclip2-HC2-S Temperature and Relative Humidity Probe. Temperature ranges from −50 °C to +100 °C (−40 °C to +60 °C, 0–1 V outputs); Relative Humidity 0% to 100%. Accuracy ±0.8% RH, ±0.1 °C.
- Standard Hygroclip2-HC2-S3 Temperature/Relative Humidity probe for meteorology applications. Temperature ranges from −50 °C to +85 °C; Relative Humidity 0% to 100%. Accuracy ±1% RH, ±0.3 °C.
- KIPP Model CMP3 pyranometer ISO second class. Spectral range 310–2800 nm (50% points), response time < 18 s (95%), non-linearity < 2.5% (0–1000 W/m^2^), directional error < 20 W/m^2^ (80° at 1000 W/m^2^), sensitivity 7–20 CV/W/m^2^ (variation < 5% from −10 °C to +40 °C), operating temperature −40 °C to +80 °C. Thermopile detector.
- MiniAir64 Mini, probe anemometer, air velocity range 0.5–40 m/s, accuracy ±0.5% of full scale, 1.5% of reading, power supply 9–24 vDC, O/P 4–20 mA. Temperature −65 °C to 150 °C.

### 3.2. Data Reception System

As mentioned above, the GPRS GRD data can be viewed on the web in graph or table format, and downloaded as a CSV file. The web interface provided a control panel to configure a personalized view showing the data of all the sensors remotely. Data were collected for the full cycles of 2012 and 2013, though it was mainly 2013 data that were analysed, with data captured every day and at every half hour.

## 4. Fixing the Sensors during the Piercing of the Wall

**Figure 8:**
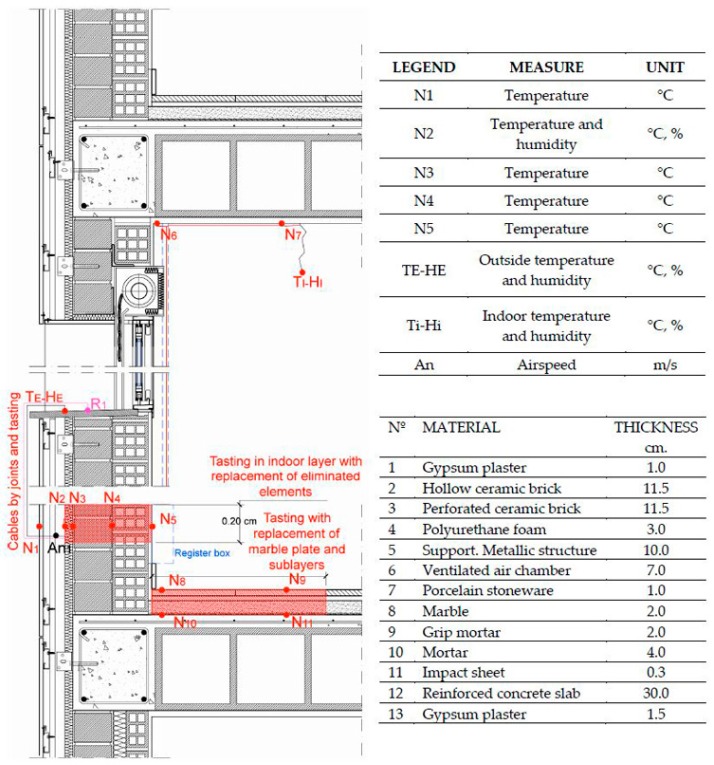
Chart of sensor locations.

**Figure 9:**
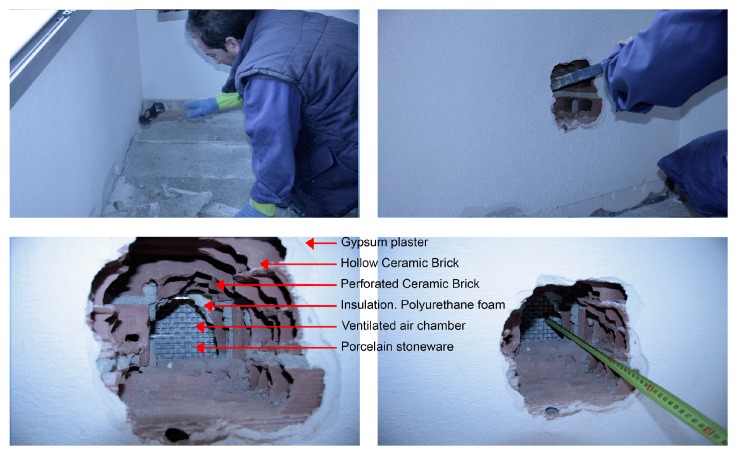
Destruction process of the enclosure to introduce the sensors.

**Figure 10:**
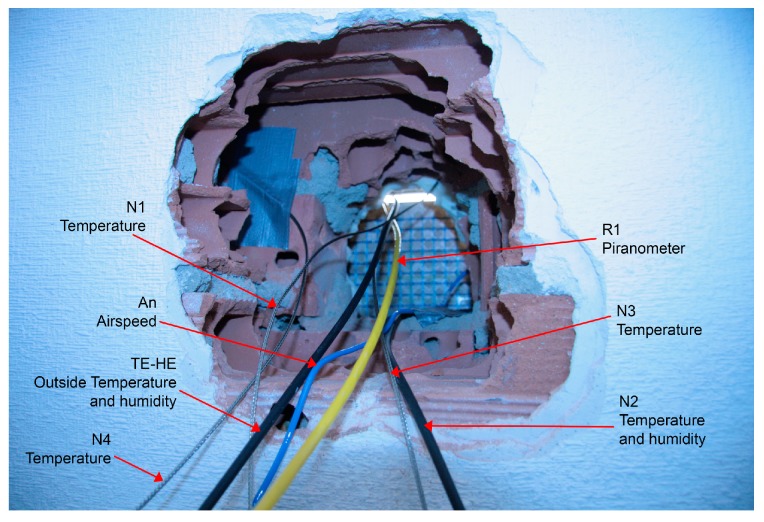
Location of sensors in the enclosure layers.

As indicated, the sensors were introduced several years after the completion of the building. After opening the enclosure, the sensors were fixed to the surfaces according to the location plan shown in Figure 8. Sensors for surface temperatures were arranged in an ordered way for the different layers at similar position coordinates (Figure 9 and Figure 10).

**Figure 11:**
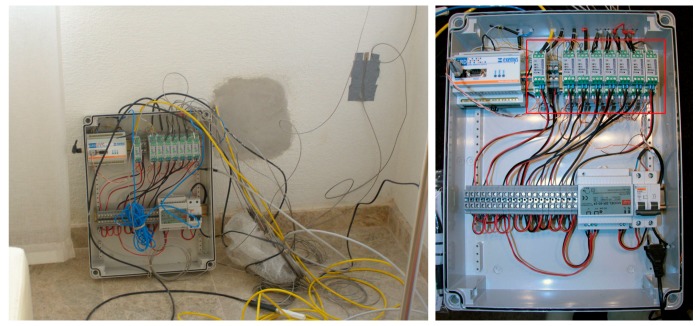
Data Logger, Data Reception and GPRS Communication.

The damage to the enclosure was repaired once the sensors were placed with connecting cables to the analyser. The various layers of plaster, hollow brick, perforated brick, polyurethane, etc., were sealed so as to ensure maximum continuity with the original materials (Figure 11). Nevertheless, for reasons of formats, shapes of the cracks, new binder materials, etc., certain discontinuities occurred in the materials, which led to differences in thermal conductivity, heat capacity, specific heat and effusivity.

## 5. Studying Heat Flow Behaviour according to Fourier

We proceeded to the calculate the enclosure’s theoretical temperature gradients based on the usual values of thermal conductivity *λ* of the materials used in the enclosure, according to UNE EN ISO 10456:2012 [22,23], and comparing them to the results of measurements carried out at the University of Alicante, of real samples, extracted by piercing the enclosure, using a thermal conductivity analyser C-Therm TCi with a universal sensor from Mathis Instruments Ltd. Calculations were performed in ideal conditions for both databases, according to Fourier’s law. The values of thermal conductivity of the samples extracted when the enclosure was pierced are shown in Table 3.

**Figure 12:**
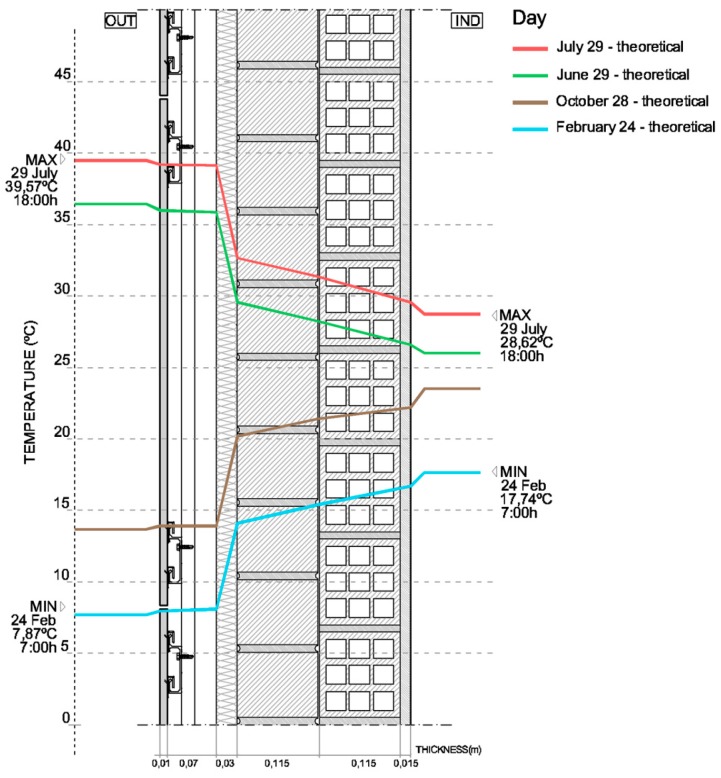
Dynamic temperature graphs of the enclosure measured according to Fourier’s law, for *λ* values of the Mathis TCi unit and thickness of the project layers, on the less favourable days in each season.

To simulate this behaviour in static conditions, unaffected by other energy sources such as solar radiation, the indoor air conditioning system, materials’ effusivity or thermal inertia [24], we applied the maximum temperature of outdoor summer air (39.57 °C), and the minimum outdoor winter temperature (7.87 °C) that had been collected by the monitoring sensors, and an indoor air temperature of 28.62 °C in summer and 17.74 °C in winter without switching on the conditioning system. Temperature gradients for the most unfavourable days of each season results are shown in Figure 12. To do this in accordance with Spanish CTE regulations [25,26], we took into account a surface thermal resistance of 1/*h_i_* of 0.13 m^2^ °C/W, a surface thermal resistance of 1/*h_o_* of 0.04 m^2^ °C/W and a thermal resistance *R_c_* of the ventilated façade cavity of 0.176 m^2^ °C/W, considered to be a poorly ventilated cavity. The reason for this choice of value was that ventilation hole surfaces represented less than 20 cm^2^ per vertical section linear meter. Furthermore, the lower part was sealed and thus closed to the passage of air. These conditions reduced the efficiency of heat dissipation in summer and the insulation layer also had a drying effect in the rainy season.

Table 4 shows the *U-value* according to the measurement values of thermal conductivities using Mathis TCi equipment, and layer thicknesses according to the project. As we will see, the temperature gradients thus obtained were significantly different from those obtained by monitoring.

## 6. Enclosure Temperature Gradients Obtained by Monitoring

As indicated, monitoring data were collected every half hour during the complete cycle of 2013. Therefore, the thermal gradients could be represented in a dynamic regime, according to the real behaviour of the enclosure, every half hour. In order to evaluate this thermal behaviour, the monitoring data were selected: moments of the year that showed the greatest difference between indoor and outdoor air temperature were chosen for each season in the year. The results of *T_i_*, *T_o_* and thermal gap Δ*T* are shown in Table 5.

**Figure 13:**
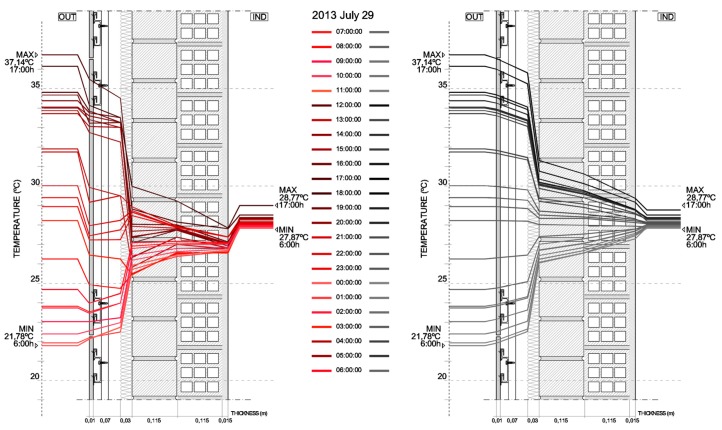
Dynamic temperature graphs showing each hour on 29 July 2013. (**Left**) Data obtained by monitoring; (**Right**) gradients in steady state according to Fourier.

**Figure 14:**
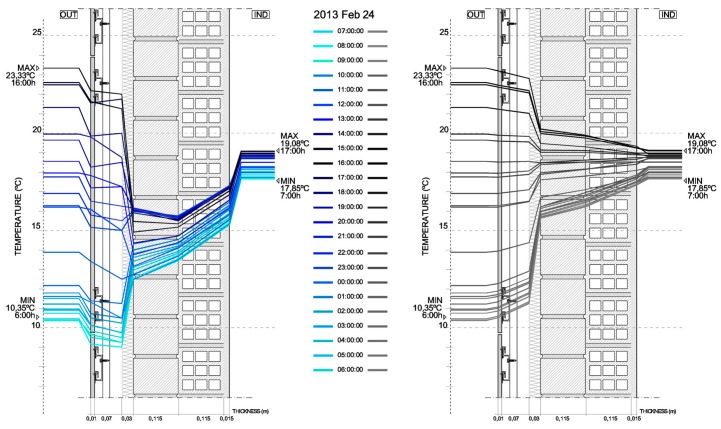
Dynamic temperature graphs by hours for 24 February 2013. (**Left**) Data obtained by monitoring; (**Right**) gradients in steady state according to Fourier.

We proceeded to study the evolution of the temperature gradients during the cycle of a full day on the four days indicated above. The results are shown in Figure 13 and Figure 14. When comparing with the graphs based on Fourier’s ideal regime, it is possible to detect, thanks to the real monitoring data, the effects on thermal behaviour produced by: the physical properties of real—not ideal—enclosures; the effect of solar radiation on the porcelain stoneware façade; the materials’ thermal inertia or effusivity; and the air conditioning systems used [27]. To this end, Figure 12 shows a comparison between graphs obtained based on Fourier for these maximum thermal gaps and graphs obtained from monitoring data.

Subsequently a metric study of thermal flow in the opaque enclosure was also carried out in order to obtain the real measurement of *U-value* [28,29]. It was measured in the two façade units (living room at a height of 1.70 m and adjoining kitchen, C1 monitoring), and also in the centre of the repaired hole (monitoring C2). For this, the ISO 9869-1:2014 standard was followed [30]. The equipment consisted of a thermal flow plate and a transducer that generates an electrical signal proportional to the total heat rate applied to the surface of the sensor.

**Scheme 1:**
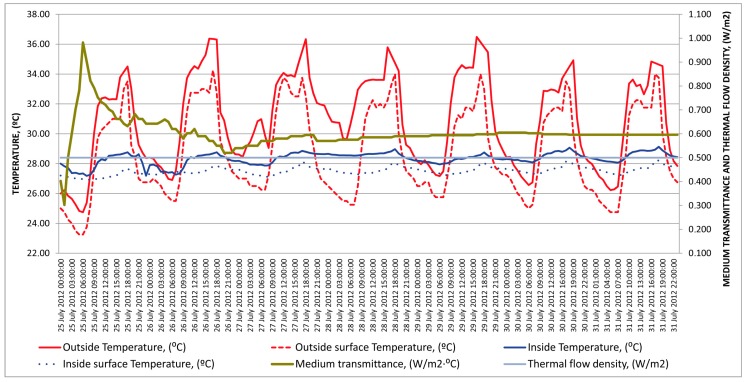
Graph of metric study of thermal flow showing the temperature and flow data from which the *U-value* was calculated.

Data were analysed using the thermal transmittance calculation module, which is part of the AMR WinControl software developed by Ahlborn for ALMEMO measuring equipment. The method used was the “average method” [31]. The results are shown in Scheme 1 and Table 6. The C1 and C2 monitoring values were higher by 10.4% and 25%, respectively, compared to the theoretical values.

**Figure 15:**
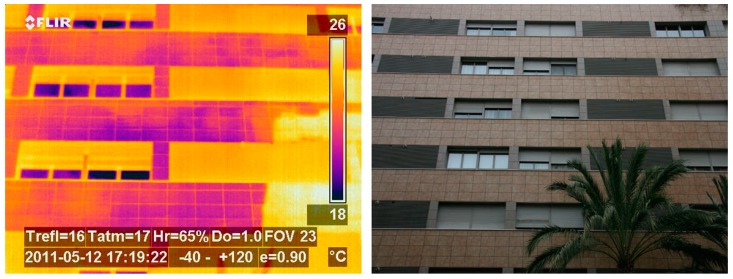
Thermographic picture of the northwest façade. Taken on 12 May 2013 at 3:55 p.m.

The heat flow analysis was completed using thermographic images, taken with ThermaCam P25 thermographic camera from Flir. We proceeded to detect thermal bridges and evaluated the behaviour of the outer enclosure in its ventilated chamber [32,33]. Quantifying thermal bridges was not, however, the aim of this research [34] (Figure 15).

## 7. Correcting Temperature Results Obtained by Monitoring

The temperature data obtained for the different layers of the enclosure did not correspond exactly to the data that would have been collected had the enclosure not been damaged or perforated to place the PT100 sensors. The subsequent repair of the layers of materials did in fact interfere, leading to material discontinuities. This was caused by three factors:

- Difficulties in execution due to the geometrical irregularity of the holes made.
- The use of a higher percentage of plaster or cement mortar.
- Difficulties in sealing the polyurethane insulation because it was impossible to access this specific layer from the outside.

The thermal conductivity *λ* values of the different layers of the enclosure shown above in Table 7 were readjusted after performing heat resistance calculations adjusted to the obtained transmittance value. Table 5 shows the comparative values of those obtained according to Fourier, using Mathis TCi data from the laboratory, the actual values obtained by monitoring, and the values that would have been obtained if the continuous enclosure had not been damaged to introduce the sensors.

Once these values of thermal conductivity *λ* were determined, the temperature data obtained by monitoring were corrected by applying a mathematical model or algorithm designed specifically for this research [34,35,36]. To address the problem stemming from changes to wall layer materials and discontinuities due to difficult technical execution or implementation, it was linked to the surface of the generated cavity, its location in the wall, and values *λ* and *λ*_1_ of the various original as well as repair materials. 

**Figure 16:**
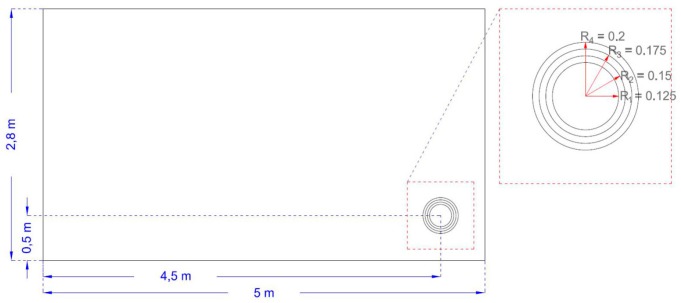
Image that shows the dimensions of the complete wall and a graphic representation of the hole synthesized in a circle of known radius.

The broken surface of each of the layers had an irregular shape that was given a simplified circle shape of a similar size and a known radius (Figure 16). The inner layers were larger than the outer ones, leading to:

$$r_{1}<r_{2}<r_{3}<r_{4}.$$

Based on this premise, the intention was to develop a system capable of interpreting the percentage of error in each of the measurements in order to eliminate it later. These measurements were different for each of the layers of the enclosure as well as over time, according to the physical laws of energy transmission. The approach consisted of responding to error coming from the impact of a circle (of radius *r*) inside a rectangular wall measured by (*w*, *y* and *h*). Using this data, a system based on two coordinate axes was set up, where the perforated circle was placed in the position (*Ax* and *Ay*) and the sensor in the position (*Bx* and *By*), corresponding to its location in each layer.

**Figure 17:**
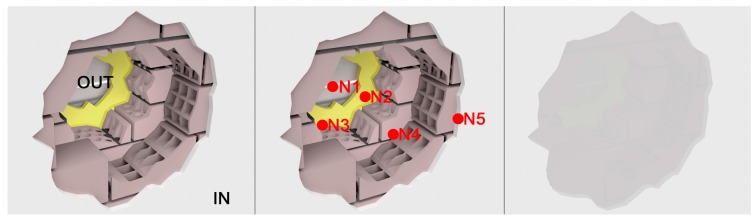
Picture of hole in the wall and subsequent repair using a different material.

By introducing the sensors after the building was constructed, it was necessary to repair the hole that had been drilled to make the measurements (Figure 17). Therefore, the punctual thermal conductivity *λ* in a wall layer, within an area where there had been no repairs, was affected by the theoretical conductivity *λ*_1_ of the material used in its execution, depending on the distance between the location of the sensor and the location of the area built with new materials (the greater the distance, the lesser the impact). That is, to correct the produced deviation, we had to quantify the thermal conductivity value *λ* for each of the layers according to the extent to which the area was affected by the sensor that had been introduced. This way, by finding the deviation ratio (global effect), it becomes possible to deduce the temperature in cases where no damage was caused by introducing the sensors.

The theoretical thermal conductivity *λ* was corrected using the generated error parameter (correction factor *f_c_*), which took into account the thermal conductivity of the new material, the proportion of repaired wall surface and the distance between the repair area and the sensor.

$$z\left(x,y\right)=\lambda +\left(\lambda_{1}-\lambda \right)*\frac{ag}{smax}*\frac{r}{\sqrt{\left\{{\left(Ax-x\right)}^{2}+{\left(Ay-y\right)}^{2}\right\}}}*\alpha$$

$$global\_effect=\int_{0}^{h}\int_{0}^{w}z\left(x,y\right)\;dx\;dy$$

$$f_{c}=\frac{\lambda }{z\left(x,y\right)}$$

The parameters of the equation are:

- Double integral of the *x* and *y* coordinates of the *z* value (correction algorithm): global_effect
- Interior wall size (*w*, *h*)
- Coefficient *λ* of the original construction material
- Coefficient *λ*_1_ of the repair material
- Centre (*Ax*, *Ay*) and radius of the cavity *r*
- Total surface area: *smax* = *wh*
- Circular hole area: *ag* = π*r*^2^
- Regularization value: *α*

A practical example could be the data of the measurement that was made:

- Size of the interior wall (500,280)
- Coefficient *λ* of the original construction material = 0.4
- Coefficient *λ*_1_ of the repair material = 0.56
- Centre (300,100) and radius of the cavity = 20
- Regularization value: *α* = 1

**Figure 18:**
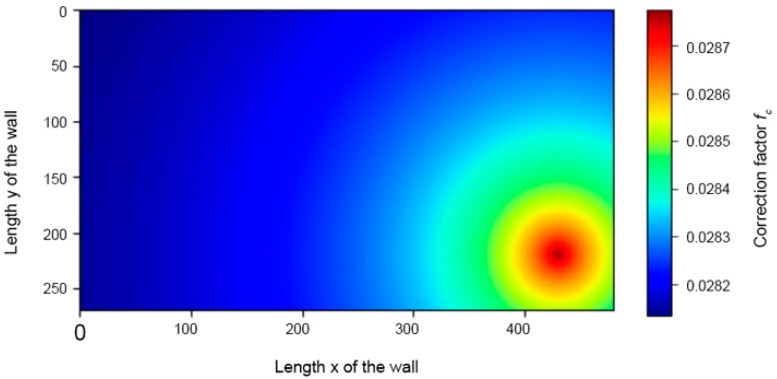
Picture showing the size of the complete wall and cavity made in each of the layers interpreted as a circle for simplification.

Taking these data into account, it is possible to visualize the results obtained after modifying the thermal conductivity *λ* of each of the points in the plane. Figure 18 shows the application of this algorithm to the interior wall of the home’s living room where the sensors were located. Table 8 shows the correction factor value for each of the sensors inserted in the inner layers of the enclosure.

**Figure 19:**
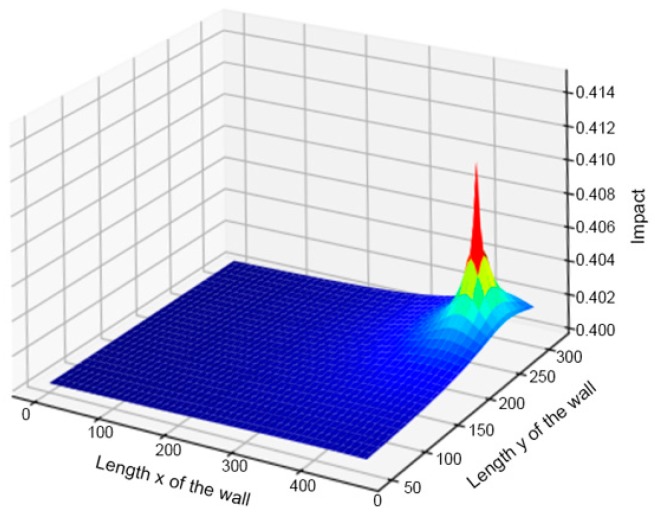
Flow diagram showing the impact of the change of materials used in an area of each of the points of the wall.

**Scheme 2:**
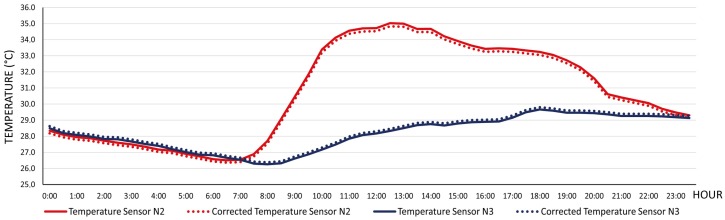
Temperature values recorded by sensors N2 and N3 on 10 August 2013. Temperature values corrected with *f_c_*.

**Figure 20:**
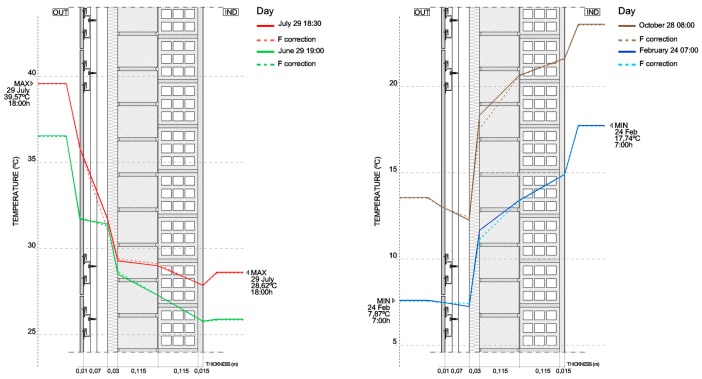
Comparison of temperature gradients before and after applying the *f_c_*.

The three-dimensional diagram (Figure 19) shows an increase in the heat flow inside the hole due to the increase in the materials’ thermal conductivity *λ*. The correction factor *f_c_* was applied to the system receiving the sensors’ surface temperature data. Data already corrected were thus visualized and entered in the database, so that the actual behaviour of the enclosure could be processed later. The actual temperature gradients experienced by the building in dynamic regime were thus obtained [37]. Scheme 2 shows the corrected temperature data for 29 July 2013, from sensors N2 and N3. The temperature gradients obtained before and after the application of the correction factor *f_c_* are shown in Figure 20, on days presenting the greatest thermal gap for each season in 2013: 24 February, 29 June, 29 July and 28 October.

**Figure 21:**
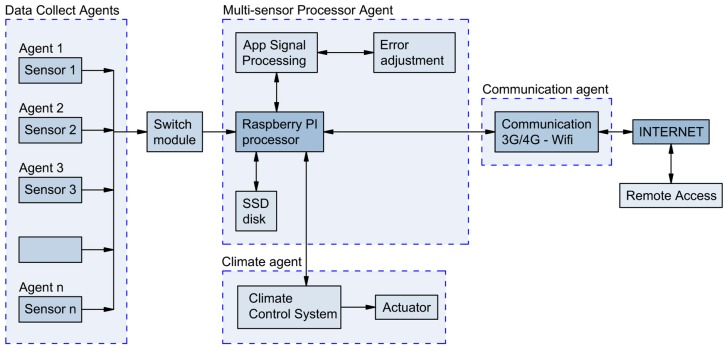
Outline of the architecture of the Multi-sensor Processing System.

The architecture designed to process the temperature data corrected by factor *f_c_*, obtained from the multiple sensors described, allowed us to improve the initial proposal by using Multi-agent System (MAS) techniques. These techniques incorporate a set of agents that distribute the required components’ different tasks so that sensor data are processed on site (Figure 21). The MAS is a field of research that covers a wide range of applications in smart buildings, construction, building sensing and control, or maintenance [38,39,40]. A modern approach to architectural construction considers that MAS components are connected to a building’s architectural design. From this perspective, a multidisciplinary approach is essential in constructive design [41], where models for Building Information complemented with MAS turn into powerful systems for holistic building design [42].

**Figure 22:**
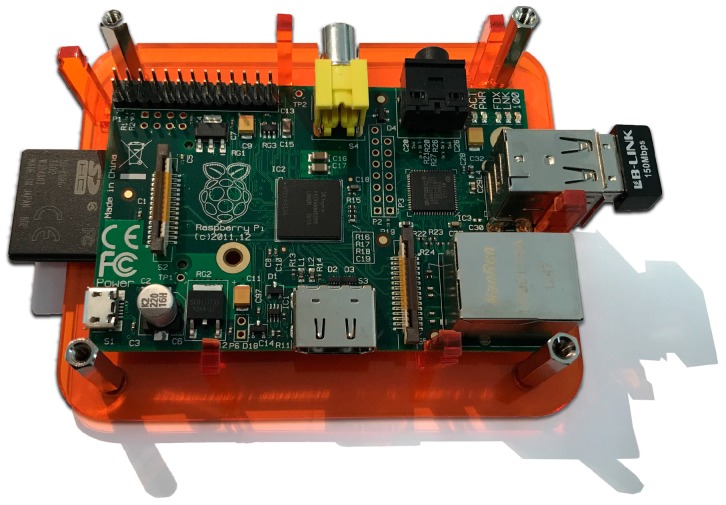
Raspberry Pi processor connected to the SSD subsystem.

The Data Collect Agents gather data from each independent sensor, connected through a switch to a Raspberry Pi processor [43] that works on a Linux operating system. The second type, the Multi-sensor Processor Agent, integrates the Raspberry Pi processor connected to an SSD subsystem for data storage and the Signal Processing Application (Figure 22). The developed app includes error adjustment, which facilitates the filtering and elimination of errors since it integrates the algorithm proposed in the article to rectify the data collected by sensors. The Communication Agent is connected to the Processor module and manages a 3 G/4 G or Wi-fi communication module. This allows for accessing the system remotely via the Internet using IP connections to communicate via Remote Access, whereby information can be accessed, monitored and visualized. Additionally, the system could be improved using a subsystem (Climate Agent) that would allow for controlling the interior climate of the house by sending orders to the physical control mechanism through an Actuator. This subsystem leads to intelligent air conditioning of the building in accordance with generated outdoor thermal changes. The algorithm implemented in Raspberry Pi processed the information from the different sensors, applying the error correction formula explained at the beginning of this section. The main algorithm correction formula was as follows:

- for *y* in range(*h*):
- for *x* in range(*w*):
  - *z* = lambda1 + lambda2 * (*ag*/*smax*) * (*r*/(100 + np.power(pow((*Ax* − *x*),2) + pow((*Ay* − *y*),2), 1/2))) * constant
  - m[*y*,*x*] = *z*
  - lostenergy = lostenergy + *z*
  - npoints = npoints + 1

**Figure 23:**
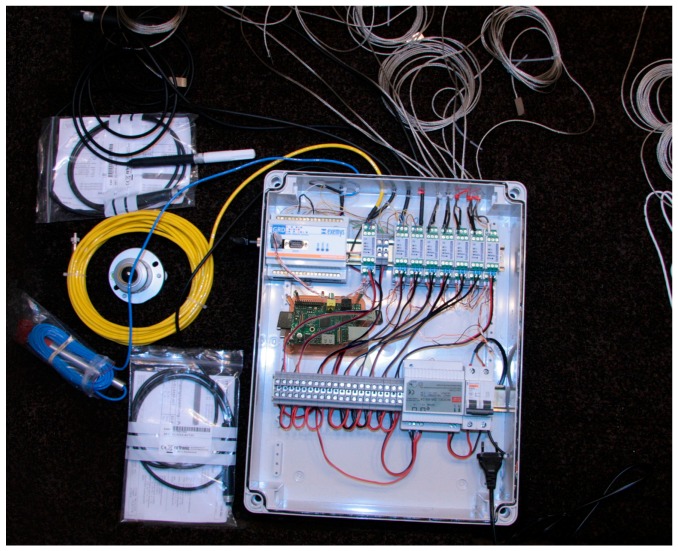
Image of the assembly of the system, with the Data Logger, the Raspberry Pi Processor and the GPRS Communication.

The working architecture of the main agents of the system is represented in Figure 23, structured in four types: Data Collet Agents, Multi-sensor Processor Agent, Climate Agent and Communication Agent.

## 8. Discussion: Comparative Analysis of Results

**Figure 24:**
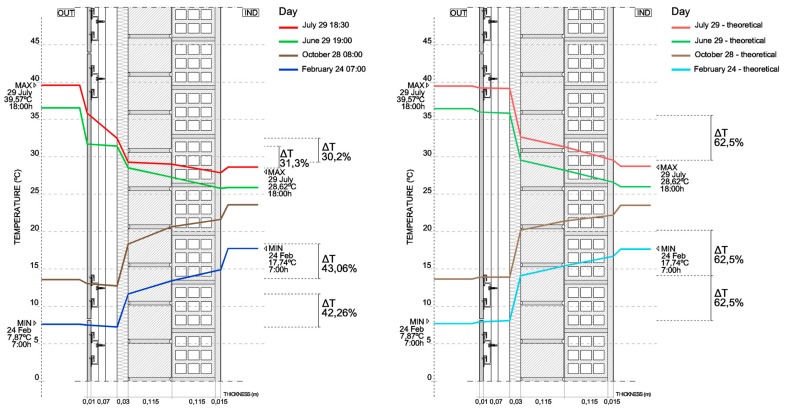
Comparison of temperature gradients in the insulation layer, and percentage of thermal gap *T_i_* − *T_o_* (**Left**) according to monitoring; (**Right**) according to the Fourier stationary regime.

Conclusions can be drawn by analysing the enclosure’s behaviour in static regime or according to Fourier’s law, compared to the actual behaviour obtained by monitoring and subsequent correction of results [44]. The thermal resistance obtained by monitoring was lower than the thermal resistance obtained via theoretical calculations or Fourier’s mathematical model. The differences were considerable. Table 9 shows the values relating to the thermal gap between both sides of the polyurethane foam insulation, and the temperature gaps between indoor and outdoor air temperature, on selected days, as well as the percentages of the comparative values. Average values of the values collected every half hour were calculated in order to mitigate the effect of thermal inertia in this comparative quantification. As shown, the thermal gap in the insulation, according to monitoring results, varied between 30.2% and 43.06% of the thermal gap *T_i_* − *T_o_*. However, in the mathematical model based on ideal conditions, this thermal gap was 62.5%. This thermal jump obtained by monitoring is 25% higher in autumn and winter compared to that obtained in summer. This greater thermal jump is due to the greater thermal jump of the outside temperature *T_o_*, in the cycle of a full day, registered during October to May. Figure 24 shows these gradients and thermal gaps.

These data corresponding to actual thermal behaviour led to three possible hypotheses to explain these significant differences:

- The thermal conductivity value *λ* of the polyurethane foam was erroneous.
- The enclosure was constructed with considerable errors of discontinuity.
- The average real thickness of the insulation was not 3 cm, but a little lower.

The first hypothesis could be discarded thanks to measurements and thermal characterization of the materials performed at the University of Alicante (Table 3). The second hypothesis was discarded when checking construction site management documentation that included photographs and reports proving that the polyurethane foam had been sprayed correctly. Undoubtedly, the third hypothesis was the most credible. We proceeded to calculate what the actual thickness of the insulation layer had to be based on monitoring data and applying the mathematical model [45], to obtain a percentage of the thermal insulation gap equal to 40.7% of the total gradients obtained for the full year cycle of 2013, corresponding to the *U-value* equal to 0.596 W/m^2^ °C measured on site according to C1 monitoring [46] (Table 6). The result, as shown in Table 10, was 2.5207 cm. As shown, the actual behaviour of the enclosure, due to the lesser efficiency of the insulation layer because of its thinness, determined the passage of the thermal wave through the enclosure. This reduces the comfort indoors, and increases the annual energy demand [47,48]. This thickness coincides with the measurement obtained in the rupture of the enclosure, which was 2.5 cm. It is not possible to know the average thickness of said polyurethane foam layer projected on the envelope assembly, but the measurement results of the *U-value* at various points confirm that this is the actual thickness, compared to 3 cm planned in the project [49].

Another conclusion was the beneficial effect on thermal comfort produced by both the porcelain stoneware and the ventilated cavity in their capacity to dissipate radiation heat in summer [50]. The obtained monitored temperature on the outer face of the polyurethane insulation, at the time of greatest thermal gap *T_i_* − *T_o_* was 8.5 °C lower than that of the porcelain stoneware; had this ventilated cavity not existed, the thermal gap would have been only 2.8 °C. That is to say, according to heat flow simulations in the Design Builder tool, the heat gain by transmission to the interior of the house would have been greater, and the indoor air temperature *T_i_* would have been 1.2 °C higher. This would have generated an 18% increase in energy demand. The effect of thermal insulation and thermal inertia of 22 cm of hollow and perforated ceramic brick can also be observed in Figure 13 and Figure 14. They cushion the thermal wave due to the produced Δ*T* of indoor–outdoor air.

## 9. Conclusions

In order to understand the thermal behaviour of existing buildings, it is not sufficient to monitor multi-layer enclosures by introducing temperature probes of indoor/outdoor air and to interior and exterior surfaces of the building. The differences in the value of thermal conductivity *λ* of the real materials compared to that of databases based on Spanish CTE regulations, as well as flaws during the construction process, or variations in the thickness of insulation such as polyurethane foam, significantly influence the thermal gradients produced and the *U-value* of thermal transmittance. In addition, the effects of thermal conductivity parameters and heat capacity of different materials making up the enclosure, or the effects of solar radiation on the façade’s surface coat, in the dynamic process of heat transmission, produce significant variations in the generated thermal gradients compared to the linear behaviour established by Fourier’s law.

In the present study, this analysis of the actual behaviour of the enclosure regarding heat flow was possible thanks to the destructive monitoring system we used. PT100 sensors were introduced in all surfaces of the layers of the multi-layer enclosure, and corrections were made to the measurements of the surface temperatures using a mathematical model designed specifically for this study. To do this, part of the enclosure had to be destroyed through piercing, the sensors were introduced in the surfaces to be measured, and the different layers were then repaired using materials similar to the original ones. These repairs caused discontinuities in the value of the thermal conductivity of the original materials of between 19.1% and 42.8%. Therefore, the temperatures measured by the sensors had to be corrected through mathematical modelling. This model took into account the geometry of the façade and the perforation made, the distance between the sensor and the centre of the hole, the distance to the limit of the change of repair material, and the thermal conductivity values *λ* and *λ*_1_. Temperatures obtained by the sensors had correction values between 0.12% and 0.46%.

The actual values of temperature gradients obtained by monitoring, once corrected, showed substantial differences to those obtained in Fourier law’s static regime. These differences are mainly due to the effect of materials’ thermal inertia during heat transmission in dynamic regime, as well as to the overheating of the porcelain stoneware layer of the façade. There is a continuous variation of the *T_o_* in the daily cycles, and significant solar radiation impact, mainly in summer, starting at 18:00 h.

Once we determined the moments in the daily cycle at which the temperature gradient collected by the sensors resembled the static regime, the thermal gap Δ*T*_3_ between both sides of the thermal insulation was found to be substantially different from the theoretical value obtained according to Fourier’s theoretical gap. It represented between 30.2% and 42.26% of the total thermal gap, whereas an estimate based on Fourier’s method produced 62.5%. Out of the three possible hypotheses, it was shown that the decrease in *R*_3_ thermal resistance was due to the fact that the actual thickness of the polyurethane foam insulation was 2.5207 cm instead of 3 cm. This is one of the key contributions of this research.

A system for correcting temperature data using Raspberry Pi was implemented in the remote sensing system, which allowed for evaluating the real impact of thermal inertia and solar radiation on the multi-layer enclosure. It will be possible, in future studies, to quantify the thermal gap’s evolution in the dynamic process of each night and day cycle and then translate it into energy savings or reduction of annual energy demand, using the Design Builder tool, which uses the energy-plus calculation engine. Five buildings in the same climate zone were monitored in a similar way, applying different enclosure construction solutions. In future research, the behaviours of these enclosures could be analysed and compared, with the aim of improving the energy efficiency of buildings on the Mediterranean coast. These future studies would allow for obtaining accurate evaluations of the impact of enclosures on buildings’ annual energy demands.

## Figures and Tables

**Table 1:** *U-value* according to *λ* values extracted from the UNE EN ISO 9869-1:2014 standard established by the CTE.

Vertical Enclosure and Horizontal Flow		Thermal Resistance		
Layers		Thickness (m)	*λ* (W/m K)	*R* (m^2^ K/W)
1	Outdoor surface resistance (O_sr_)			0.040
2	Discontinuous stoneware cladding	0.010	2.300	0.004
3	Poorly ventilated cavity	0.070		0.176
4	Sprayed polyurethane thermal insulation	0.030	0.023	1.304
5	Perforated ceramic brick	0.115	0.760	0.151
6	Hollow ceramic brick	0.115	0.490	0.235
7	Plaster layer and coat	0.015	0.300	0.050
8	Indoor surface resistance (I_sr_)			0.130
*R_T_* = Suma *R_i_* (m^2^ K/W)				2.101
*U_T_* = 1/*R_T_* (W/(m^2^ K))				0.476

**Table 2:** General organization of the sensor system to monitor type C flat.

Field Equipment (Indoor and/or Outdoor)	Building 1 (Alicante)
Outdoor environment humidity–temperature sensor	1
Indoor environment humidity–temperature sensor	1
Interstitial humidity–temperature sensor	2
Solar radiation sensor	2
Wind speed sensor	1
Temperature sensor	23
TOTAL	30

**Table 3:** Values of thermal conductivity *λ* according to the UNE EN ISO 6946:2012 standard and measurements taken using Mathis TCi equipment.

Enclosure Layer Materials	Thickness *t* (cm)	Thermal Conductivity *λ* (W/m °C)	Thermal Conductivity *λ* Mathis TCi (W/m °C)
Plaster coat layer	1.0	0.3	0.27
Hollow ceramic brick	11.5	0.49	0.52
Perforated ceramic brick	11.5	0.76	0.75
Insulation. Polyurethane foam.	3	0.023	0.028
Poorly ventilated cavity	1.0	2.30	2.21
Porcelain stoneware pieces	1.0	0.3	0.27

**Table 4:** *U-value* according to *λ* values obtained using Mathis TCi equipment. Percent difference of values of resistance to the passage of heat *R* with respect to Table 1.

Vertical Enclosure and Horizontal Flow		Thermal Resistance			
Layers		Thickness (m)	*λ* (W/m K)	*R* (m^2^ K/W)	Δ (%)
1	Outdoor surface resistance (O_sr_)			0.040	
2	Discontinuous stoneware cladding	0.010	2.210	0.005	+25
3	Poorly ventilated cavity	0.070		0.176	
4	Sprayed polyurethane thermal insulation	0.030	0.028	1.071	−17.87
5	Perforated ceramic brick	0.115	0.750	0.153	+1.32
6	Hollow ceramic brick	0.115	0.520	0.221	−5.96
7	Plaster layer and coat	0.015	0.270	0.056	+12
8	Indoor surface resistance (I_sr_)			0.130	
*R_T_* = Suma *R_i_* (m^2^ K/W)				1.852	−11.85
*U_T_* = 1/*R_T_* (W/(m^2^ K))				0.540	+13.45

**Table 5:** Results for *T_i_ − T_o_* during the greatest thermal gaps for each climatic season.

Enclosure Layer Materials	Day	Time	*T_i_* (°C)	*T_o_* (°C)	Δ*T* (°C)
Spring	29 June 2012	19:00	25.22	36.95	11.73
Summer	29 July 2012	18:30	28.62	39.57	10.95
Autumn	28 October 2012	08:00	23.36	13.92	9.44
Winter	24 February 2013	07:00	17.74	7.60	10.14

**Table 6:** Results of *U-values* across different scenarios.

	Fourier UNE	Fourier Mathis TCi	Monitoring C1	Monitoring C2
*U* (W/m^2^ °C)	0.476	0.540	0.596	0.675

**Table 7:** Results of *U-value* across different scenarios.

Enclosure Layer Materials		Thermal Conductivity *λ* W/m °C	Thermal Conductivity *λ* Mathis TCi	Thermal Conductivity *λ* Monitoring	Thermal Conductivity *λ* Repair
1	Plaster coat	0.30	0.27	0.25	0.27
2	Hollow ceramic brick	0.49	0.52	0.48	0.62
3	Perforated ceramic brick	0.76	0.75	0.69	0.85
4	Insulation. Polyutherane	0.023	0.028	0.025	0.040
5	Porcelain stoneware	2.30	2.21	1.89	2.21

**Table 8:** Results of *U-value* across different scenarios.

Materials of the Enclosure Layers	Thickness *t* (cm)	Ref. Sensor	Correction Factor *f_c_ T_i_ < T_o_*	Correction Factor *f_c_ T_i_ > T_o_*
Plaster coat	1.0	N5	1.0012	0.9988
Hollow ceramic brick	11.5	N4	1.0024	0.9976
Perforated ceramic brick	11.5	N3	1.0046	0.9954
Polyurethane foam	3	N2	0.9945	1.0055
Poorly ventilated cavity	7.0	N1	1	1

**Table 9:** Results of *T_i_* − *T_o_* of the insulation layer during the greatest thermal gaps per climate season. Percentage according to total thermal gap.

Days with Greatest Thermal Gap per Season	*T_i_* °C	*T_o_* °C	Δ*T* °C	*T_i_* Insulation	*T_o_* Insulation	Δ*T* Insulation	%
24 February 2013 07:00:00	17.74	7.60	10.14	11.92	7.15	4.77	47.04
29 June 2012 19:00:00	25.22	36.95	11.73	28.15	32.25	4.10	34.95
29 July 2012 18:30:00	28.62	39.57	10.95	29.20	32.82	3.62	33.06
28 October 2012 08:00:00	23.36	13.92	9.44	17.09	12.55	4.54	48.09

**Table 10:** Value of the actual thickness of the thermal insulation of polyurethane foam, according to *λ* obtained by the Mathis TCi equipment, and *U-value* according to C1 monitoring.

Vertical Enclosure and Horizontal Flow		Thermal Resistance		
Layers		Thickness (m)	*λ* (W/m K)	*R* (m^2^ K/W)
1	Outdoor surface resistance (O_sr_)			0.040
2	Discontinuous stoneware cladding	0.010	2.300	0.005
3	Poorly ventilated cavity	0.070		0.176
4	Sprayed polyurethane thermal insulation	**0.0252**	0.028	0.900
5	Perforated ceramic brick	0.115	0.750	0.153
6	Hollow ceramic brick	0.115	0.520	0.221
7	Plaster layer and coat	0.015	0.270	0.056
8	Indoor surface resistance (I_sr_)			0.130
*R_T_* = Suma *R_i_* (m^2^ K/W)				1.681
*U_T_* = 1/*R_T_* (W/(m^2^ K))				**0.596**
